# Functional role of MicroRNA/PI3K/AKT axis in osteosarcoma

**DOI:** 10.3389/fonc.2023.1219211

**Published:** 2023-06-19

**Authors:** Yubo Xiang, Yingxin Yang, Jia Liu, Xu Yang

**Affiliations:** Department of Rehabilitation Medicine, General Hospital of Northern Theater Command, Shenyang, China

**Keywords:** MicroRNA, PI3K/AKT pathway, osteosarcoma, mechanism, biomarker

## Abstract

Osteosarcoma (OS) is a primary malignant bone tumor that occurs in children and adolescents, and the PI3K/AKT pathway is overactivated in most OS patients. MicroRNAs (miRNAs) are highly conserved endogenous non-protein-coding RNAs that can regulate gene expression by repressing mRNA translation or degrading mRNA. MiRNAs are enriched in the PI3K/AKT pathway, and aberrant PI3K/AKT pathway activation is involved in the development of osteosarcoma. There is increasing evidence that miRNAs can regulate the biological functions of cells by regulating the PI3K/AKT pathway. MiRNA/PI3K/AKT axis can regulate the expression of osteosarcoma-related genes and then regulate cancer progression. MiRNA expression associated with PI3K/AKT pathway is also clearly associated with many clinical features. In addition, PI3K/AKT pathway-associated miRNAs are potential biomarkers for osteosarcoma diagnosis, treatment and prognostic assessment. This article reviews recent research advances on the role and clinical application of PI3K/AKT pathway and miRNA/PI3K/AKT axis in the development of osteosarcoma.

## Graphical Abstract

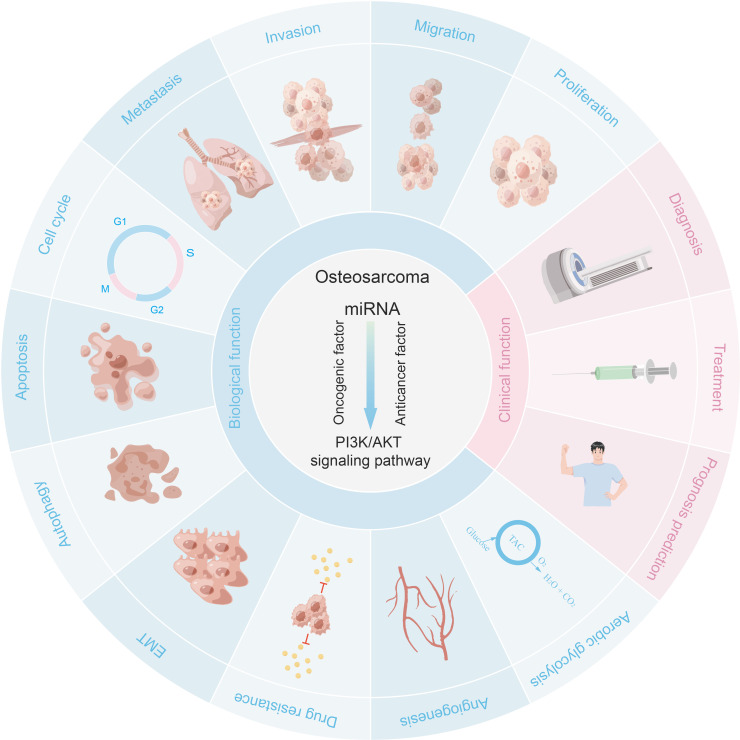

## 1 Introduction

Osteosarcoma (OS) is a primary malignant bone tumor derived from bone forming mesenchymal stem cells. It is highly malignant and can be locally aggressive and often leading to pulmonary or even systemic metastases. Children and adolescents are the most common patients with osteosarcoma, second only to lymphoma and brain tumors in the childhood and adolescent population (1–3). The distal end of the femur is the most common site for osteosarcoma, followed by the proximal end of the tibia and humerus (2, 4–6). Local invasion is observed in more than 85% of osteosarcoma patients, with lung metastases being the most common in 74% of patients with metastases, followed by bone metastases in 9% of patients, and both bone and lung metastases in 8% of patients with metastases (6). In recent decades, surgery combined with new chemotherapy has been recognized as the standard treatment for osteosarcoma, significantly improving overall survival and quality of life (7). Emerging chemotherapy regimens include cisplatin (DDP), adriamycin (DOX), methotrexate (MTX), and isocyclophosphamide (IFO) (7). However, the therapeutic effect of chemotherapeutic agents is limited by various reasons, such as escape of apoptosis, reduced drug uptake, and increased drug metabolism. Systemic metastasis limits the effectiveness of surgical resection, so metastatic and drug resistance often result in unsatisfactory outcomes and prognosis for patients with osteosarcoma (8). The problems described above involve changes in multiple biological processes, including changes in genetic and epigenetic characteristics. Understanding and studying the molecular changes of genes associated with the formation of osteosarcoma and associated signaling pathways will help uncover the mechanisms underlying its occurrence and development, providing new directions for the diagnosis, targeted therapy, and prognosis of osteosarcoma.

More than 98% of the genes in the human genome are composed of noncoding genes (9–11). Since they lack the ability to encode proteins, their transcripts are considered non-coding Rna (ncRNA) (12, 13). With the development of high-throughput sequencing technology, the characteristics of ncRNAs have gradually emerged, and microRNA (miRNA, miR), long ncrna (lncRNA) and circular RNAs (circRNA) are considered as classical ncRNA (14–16). Among them, miRNA is a conserved endogenous non-coding RNA of approximately 22 nucleotides in length (17, 18). MiRNA can bind to the untranslated region (UTR) of mRNA and regulate the expression of target genes by inhibiting the translation or degradation of mRNA, thereby affecting a variety of intracellular signaling pathways and playing an important role in the formation of tumors (19). The abnormal expression of miRNA in tumor cells can affect the malignant biological behavior of tumor cells (20–23), and osteosarcoma is no exception. MiRNA have a very important role in the development of osteosarcoma and are clearly associated with many clinical features (24–27). MiRNAs are expected to become effective biomarkers for diagnosis, targeted therapy and prognosis of osteosarcoma patients.

It is well known that the PI3K/AKT pathway plays an extremely important role in the life activities of cells (28), It can be activated by insulin growth factors and cytokines under physiological conditions and participates in the regulation of a variety of intracellular signal transduction and cell biological processes (29), such as cell growth, differentiation, transcriptional regulation, protein synthesis, metabolism, autophagy, cell proliferation, apoptosis, angiogenesis, migration, and cytoskeletal reorganization (30–36). Aberrant activation of the PI3K/AKT pathway has been observed in almost all types of tumor cells (37–39), such as ovarian cancer (40, 41), lung cancer (42, 43), gastric cancer (44, 45), pancreatic cancer (46, 47), breast cancer (48, 49), hepatocellular carcinoma (50, 51), lymphoma (52, 53), osteosarcoma (54) and so on. Therefore, an in-depth study of the function of PI3K/AKT pathway in carcinogenesis is of great importance.

Recently, there is increasing evidence that the interaction between miRNA and PI3K/AKT pathway has an important role in the biological process of osteosarcoma (55–58). Moreover, this interaction has been found to be significantly associated with many clinical features (59, 60), and these studies provide a new perspective for the diagnosis, targeted therapy and prognosis of osteosarcoma patients. In recent years, the study of miRNA associated with the PI3K/AKT pathway has also been a hot spot for investigating mechanisms related to osteosarcoma development. In this review, we review the molecular mechanisms and functional roles of the miRNA/PI3K/AKT axis in the pathogenesis and progression of osteosarcoma.

## 2 PI3K/AKT pathway in osteosarcoma

It is well known that aberrant activation of the PI3K/AKT pathway may lead to tumorigenesis. In recent years, a large amount of evidence has shown that dysregulation of PI3K/AKT pathway is involved in a variety of pathological processes of OS, including OS occurrence, proliferation, metastasis, migration, invasion, cell cycle progression, apoptosis, autophagy, angiogenesis, chemoresistance, Epithelial-Mesenchymal Transition (EMT), aerobic glycolysis, etc. This section introduces the PI3K/AKT pathway and outlines the mechanisms involved in the role of PI3K/AKT pathway in the development of osteosarcoma.

### 2.1 Overview of the PI3K/AKT pathway

Abnormalities in the PI3K/AKT pathway are common in osteosarcoma, and the PI3K/AKT pathway plays a critical role in regulating the growth, proliferation, differentiation, migration, metastasis, infiltration, apoptosis, and drug resistance of osteosarcoma (61–65). It has been demonstrated that the dysregulation of major factors in this signaling pathway in osteosarcoma cells is closely related to the activation and inhibition of other downstream signaling pathways. PI3K, a member of the large family of lipid kinases, is a downstream effector of receptor tyrosine kinases (RTKs) and G protein-coupled receptors (GPCRs) (66). Based on the differences in structure and function of PI3K, it has been classified into three subclasses, class I, II and III (36, 67). Among them, class I PI3K is the most relevant to tumors, and the most intensive research has been conducted on class I. Class I is further divided into class IA and class IB, and they are composed of the p110α, β, γ and δ catalytic subunits encoded by the PIK3CA, PIK3CB, PIK3CG and PIK3CD genes, respectively, and the PIK3R1, PIK3R2 and PIK3R3 genes encoding p85α, p85β and p55γ regulatory subunits of the PIK3R1, PIK3R2 and PIK3R3 genes (68, 69). The binding between subunits not only stabilizes the structure of PI3K but also provides sites for activation of PI3K by RTKs, GPCRs, and oncogenes (e.g. Ras) (36, 67, 70). Various molecules including insulin, epithelial growth factor (EGF), glucose, fibroblast growth factor (FGF) and vascular endothelial growth factor (VEGF) can activate PI3K *via* RTKs and GPCRs (71, 72). Activated PI3K can convert phosphatidylinositol 3,4-bisphosphate (PIP2) to 3,4,5-trisphosphate (PIP3), and PIP3 can bind to phosphatidylinositol-dependent kinase 1 (PDK1) to phosphorylate AKT (73). During this process, the negative regulator phosphatase and tensin homologue (PTEN) can invert PIP3 to PIP2 to limit the intensity of this activation process (74).

AKT is a serine/threonine kinase encoded by the PKB gene. AKT can lead to the activation of the downstream PI3K/AKT pathway through the phosphorylation of various substrates, including AKT1, AKT2 and AKT3, which is an extremely important protein molecule in the PI3K/AKT pathway (75). Activation of AKT is mainly the result of PDK-1 and mTORC2 phosphorylation at threonine 308 and serine 473, respectively (76, 77).AKT can also be inhibited by dephosphorylation of CTMP, PP2A, and tcl1 (78–80), after which activated AKT is transferred to the cytoplasm and nucleus, where it can activate or inhibit matrix metalloproteinases (MMPs) through phosphorylation and dephosphorylation, cyclic-dependent kinase (CDKs), MDM2, GSK3β, FOXO1, and other downstream substrates (71, 81–83), thus affecting various cellular signaling pathways and metabolic pathways and leading to abnormal life activities in normal cells.

### 2.2 Role of PI3K/AKT pathway in OS development

#### 2.2.1 Malignant phenotype of osteosarcoma

**Figure 1:**
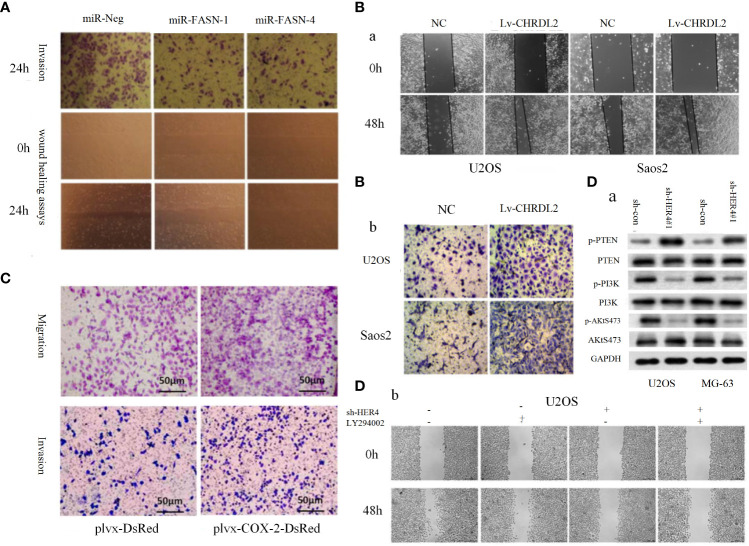
**(A)** Representative images of the transwell invasion assays and wound healing assays are shown for each group (85). **(B)** Overexpression of CHRDL2 promoted osteosarcoma cell proliferation and mobility. **(a)** Wound healing assay. **(b)** Transwell assay (86). **(C)** COX-2 overexpression increases migration and invasion in MG-63 cells (87). **(D)** The role of HER4 in the PI3K/mTOR signaling pathway. **(D-a)** The western blotting was used to measure the protein expression of p-PI3K, p-AKT, and p-PTEN. **(D-b)** Wound healing assay was performed to measure the migration ability of cells (88).

**Figure 2:**
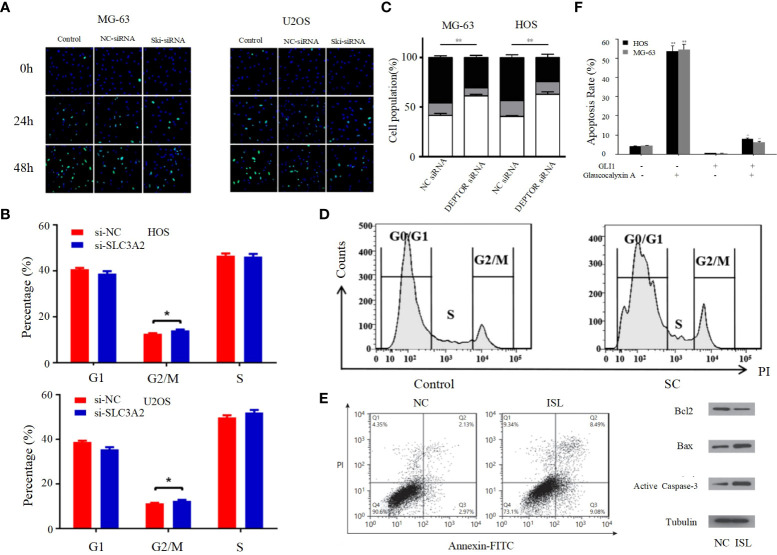
**(A)** Proliferation rates of MG63/U2OS cells among the control, NC-siRNA and Ski-siRNA groups at 24 and 48 h following transfection (62). **(B)** Cell cycle profiles determined by propidium iodide (PI) staining and flow cytometry assays of **(a)** MNNG/HOS and **(b)** U2OS cells transfected with si-SLC3A2 or si-NC (96). **(C)** Flow cytometric analysis of the percentage of cells in different phases of the cell cycle with three independent experiments (97). **(D)** Following SC treatment, cell cycle distribution was determined by flow cytometry at 24 h (98). **(E)** ISL treatment induces apoptosis in U2OS cells (63). **(F)** The apoptotic rates of HOS and MG-63 cells were detected by Annexin V/PI double-staining assay (99). *p < 0.05, **p < 0.01.

PI3K/AKT pathway and its upstream and downstream related molecules can have a significant impact on the formation of osteosarcoma and the associated malignant phenotype. This evidence strongly suggests the importance of the PI3K/AKT pathway for osteosarcoma formation, as evidenced by whole-genome sequencing analysis of OS cell lines that revealed significant upregulation of AKT expression, followed by significant inhibition of proliferation in all cell lines by the addition of the metamorphic AKT inhibitor MK-2206 (84). The levels of phosphorylated PI3K (p-PI3K) and phosphorylated AKT (p-AKT) are closely related to the activation of PI3K/AKT pathway, downregulation of fatty acid synthase (FAS) significantly reduces the expression levels of p-PI3K and p-AKT, which in turn reduced the proliferation and invasion of U-2OS cells (**Figure 1A**) (84). Chordin-like 2 (CHRDL2) is one of the Bone morphogenic proteins (BMPs) antagonist that prevents the interaction between BMPs and their cognate cell surface receptors (89). CHRDL2 is highly expressed in osteosarcoma tissues, and it was confirmed experimentally that CHRDL2 promotes the metastasis and proliferation of osteosarcoma cells through the BMP-9/PI3K/AKT pathway (**Figure 1B**) (86). Cyclooxygenase-2 (COX-2) is a membrane-bound protein closely related to inflammatory diseases. It is an inducible cyclooxygenase and rate-limiting enzyme for prostaglandin synthesis. Most tissue cells do not express COX-2 under physiological conditions, but it shows an increasing trend under pathological conditions such as inflammation and tumor (90). In osteosarcoma, COX-2 affects the expression levels of vimentin, E-cadherin, MMP-9 and MMP-2 by activating the PI3K/AKT/NF-κ b signaling pathway, leading to a significant increase in the migratory ability of osteosarcoma cells (**Figure 1C**) (87). Studies have reported that glycoprotein non-metastatic melanoma protein B (GPNMB) affects the metastasis of tumor cells in addition to being associated with tissue regeneration, inflammation, and cell proliferation (91). GPNMB may also be a potential target for targeted therapy of osteosarcoma, as it has been found that GPNMB can regulate the metastasis and proliferation of osteosarcoma cells by affecting the PI3K/AKT/mTOR pathway (92). PTEN, the first tumor suppressor gene identified with tyrosine phosphatase activity (93), is a potent negative regulator of AKT and plays a crucial role in controlling PI3K/AKT signaling activation (94). MiR-221, miR-17, and miR-128 overexpression leads to the 3-UTR of PTEN by directly binding diminished inhibition of PTEN and activation of the PI3K/AKT pathway, which significantly promotes OS cell proliferation, migration and invasion (94, 95). In a similar vein, HER4 is a member of the ErbB family, and it has been demonstrated that HER4 can promote osteosarcoma progression in part by affecting the PTEN/PI3K/AKT pathway (**Figure 1D**) (88). On the other hand, if we block the PI3K/AKT signaling cascade by various means can prevent the development of OS-related malignant phenotypes. A recent study showed that OS cell proliferation was significantly inhibited after Ski knockdown, and in-depth studies revealed a significant decrease in the protein levels of p-PI3K and p-AKT in the cells, thus the mechanism was hypothesized to be the knockdown of Ski blocking the PI3K/AKT pathway (**Figure 2A**) (62). Melanoma deficiency factor 2 (AIM2) is lowly expressed in osteosarcoma cells, and overexpression of AIM2 inhibits the levels of p-PI3K, p-AKT and p-mTOR thereby suppressing the proliferation, invasion and migration of osteosarcoma cells, a process that can be reversed by LY294002, suggesting that AIM2 is a tumor suppressor (61). However, this study lacks research on the upstream mechanism of AIM2, and no animal experiments have been conducted to further confirm this conclusion. Therefore, it has certain limitations. Molecules such as Schisandrin B (Sch B), Budding uninhibited by benzimidazoles 1 (BUB1), can affect the malignant phenotype of osteosarcoma by activating or inhibiting the PI3K/AKT pathway (100, 101).

#### 2.2.2 Cell cycle

The entire process of cell division from the completion of one division to the end of the next is called the cell cycle, and the strictly conserved cell cycle control mechanism is the main regulatory mechanism of cell proliferation; cancer is the result of continuous overdivision of cells, and therefore dysregulation of the cell cycle is closely related to the biological behavior of osteosarcoma (102). The cell cycle consists of interphase and mitotic phase consisting of G1, S and G2 phases. The transition from G1 to S phase and G2 to M phase are two very important phases of the cell cycle, which are very complex and active, and are particularly susceptible to abnormal environmental conditions thus appearing to lead to abnormal cell cycle emergence (64). Kexiang Zhang et al. recently found that Notch1 inhibited PI3K/AKT signaling, leading to S-phase block and effectively inhibiting the proliferation of osteosarcoma cells (103). Yong Cui et al. also demonstrated that S-phase block could be induced in osteosarcoma cells by altering the PI3K/AKT pathway (**Figure 2B**) (104). In addition, G2/M phase block can also be caused through the PI3K/AKT pathway, thereby inhibiting the proliferation of OS cells, as confirmed by Bin Zhu et al. (**Figure 2C**) (96). PI3K/AKT pathway can also be inhibited by activating mTOR thereby inducing G0/G1 phase cell cycle arrest in OS cell lines (97). Cyclin family members can interact with CDK proteins to form an active heterodimer complex, which is necessary for the formation of specific phases of the cell cycle. Therefore, cyclin and CDK proteins play an important role in the progression of the cell cycle (105). It has been demonstrated that the PI3K/AKT signaling pathway can influence the cell cycle progression of osteosarcoma cells by affecting the expression of cell cycle proteins and CDK. For example, DA-LIANG KONG et al. significantly inhibited the phosphorylation of AKT by sodium cantharidininate (SC) without inhibiting mTOR, JNK or p38, and inhibition of AKT phosphorylation decreased the expression of CDK4, CDK6 and cyclin D1, which induced G0/G1 phase block in MG-63 cells that further inhibited the proliferation of osteosarcoma cells (**Figure 2D**) (104). This shows that the PI3K/AKT signaling pathway has an extremely important role in influencing the cell cycle progression of osteosarcoma cells, and its in-depth study is of great significance.

#### 2.2.3 Apoptosis

**Figure 3:**
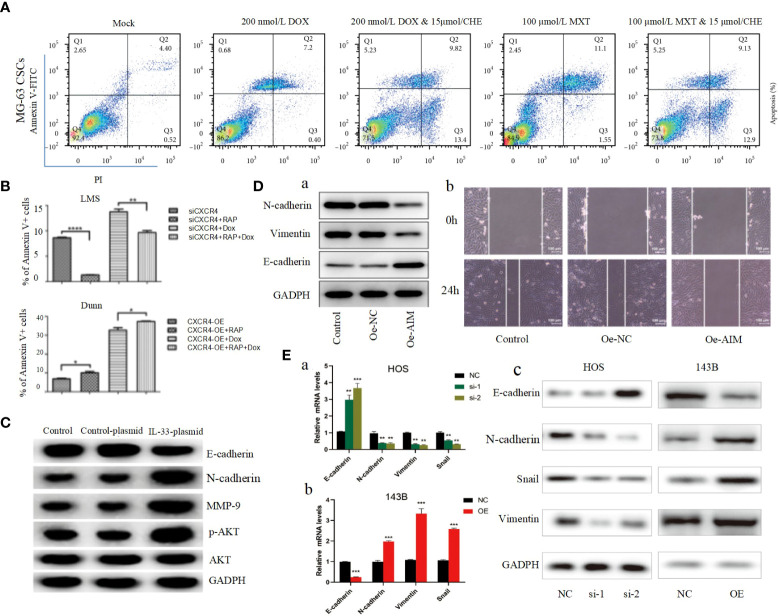
**(A)** After being cocultured using CHE with DOX or MTX, apoptosis was measured by performing Annexin V-FITC/PI double staining followed by flow cytometry assay (124). **(B)** The apoptosis ratios for each group (percentage of Annexin V+ cells) were determined by flow cytometry (125). **(C)** The protein levels of the EMT markers, E-cadherin, N-cadherin, MMP-9, p-AKT, and AKT in transfected cells were detected by Western blot (126). **(D)** Overexpression of AIM2 inhibits osteosarcoma cell invasion, migration and EMT. **(A)** Western blotting was used to assess the levels of EMT-related proteins, including N-cadherin, Vimentin and E-cadherin. **(B)** Wound healing assay was utilized to detect cell migration (61). **(E)** ZCCHC12 promoted OS cell EMT progression, qRT-PCR **(A, B)** and western blot analysis **(C)** were performed to examine EMT-related markers in OS cells after ZCCHC12 knockdown or overexpression (104). *p < 0.05; **p < 0. 01; ***p < 0.001; ****p < 0.0001.

Apoptosis, a self-destructive mechanism present in cells, has the main role of removing senescent and abnormal cells and maintaining a normal physiological state of internal environmental homeostasis. In pathological states, the homeostasis of apoptosis can be dysregulated, which can negatively affect the organism and may lead to the development of a range of tumors, including osteosarcoma (106, 107). Apoptosis is mainly initiated by the death receptor pathway, which is mediated by death receptors including tumor necrosis factor (TNF) receptors, TNF-related apoptosis-inducing ligand (TRAIL) receptors and Fas, and the mitochondrial apoptosis pathway, also known as the Bcl-2 regulatory pathway, as it is mainly regulated by the Bcl-2 family (108). Several recent studies have suggested that in osteosarcoma, abnormalities in the PI3K/AKT pathway may affect the apoptotic program of osteosarcoma cells (100, 101, 109–117). For example, Jing Chen et al. used Isoliquiritigenin (ISL) to inhibit the PI3K/AKT pathway and found that the protein expression levels of Bax and active Caspase-3 were elevated, while Bcl-2 levels were significantly decreased, and further studies revealed that apoptosis was accelerated and the invasion, proliferation and migration of osteosarcoma cells were inhibited (**Figure 2E**) (63), thus suggesting that the mitochondrial apoptotic pathway would be partially affected by the PI3K/AKT pathway. This conclusion was further supported by the use of agonists of the PI3K/AKT pathway to reverse the regulation of apoptosis and proliferation in a study by Songjia Ni et al. (118).Sineocolis homolog box homolog 1 (SIX1), an evolutionarily conserved transcription factor (119), is a key regulator of embryonic development and is associated with tumorigenesis and development (120). In osteosarcoma cells, overexpression of SIX1 inhibits apoptosis, promotes cell migration, invasion and proliferation, and in-depth studies have revealed that this function is closely related to reduced PTEN expression and activation of the PI3K/AKT pathway (121). Glaucocalyxin A has properties including inhibition of platelet aggregation (122), immunosuppressive, antioxidant and DNA damage protective activity, and cytotoxic activity (123). Recently, it was found that Glaucocalyxin A can induce apoptosis in osteosarcoma cells by increasing the ratio of Bax to Bcl-2, triggering reactive oxygen species (ROS) generation, decreasing mitochondrial membrane potential and inducing caspase-3 and caspase-9 cleavage, as found by using PI3K activators and inhibitors This function of Glaucocalyxin A is mainly achieved by inhibiting the nuclear translocation of GLI1 through the regulation of PI3K/AKT pathway (**Figure 2F**) (99). Furthermore, it has been found that inhibition of the PI3K/AKT/mTOR signaling pathway promotes apoptosis in osteosarcoma cells induced by chemotherapeutic agents, including DOX and methotrexate (MTX) (**Figure 3A**) (124). This finding has positive implications for contributing to the current challenge of chemoresistance.

#### 2.2.4 Autophagy

Autophagy is an intracellular degradation process with highly conserved characteristics. Damaged organelles and cytoplasmic proteins are encapsulated into double-layer vesicles, which then interact with lysosomes to form autolysosomes for degradation, thereby renewing cytoplasmic proteins or organelles (127, 128). Autophagy is particularly important for cells to maintain homeostasis and adapt to nutrient deficiencies *in vivo*. Three different types of autophagy include microautophagy, macroautophagy and molecular chaperone-mediated autophagy (129). There are multiple regulatory mechanisms of autophagy and the most studied mechanism, PI3K/AKT/mTOR pathway, is activated under normal nutritional conditions leading to autophagy inhibition, however, during nutritional deficiency, PI3K/AKT/mTOR pathway is inhibited leading to autophagy occurrence (129, 130). It is well known that LC3-II, ATG5 and p62 protein levels are closely related to autophagy levels, and several studies have shown that inhibition of the PI3K/AKT/mTOR pathway in osteosarcoma cells can increase the expression levels of LC3-II, ATG5 and p62, which increases autophagy and decreases the proliferative and invasive potential of osteosarcoma cells (64, 131, 132). Jinfeng Zhou et al. also found that activation of autophagic flux induced by inhibition of PI3K/AKT/mTOR signaling pathway sensitized OS to DOX (**Figure 3B**). Therefore, targeting the CXCR4/PI3K/AKT/mTOR autophagy axis may be an effective therapeutic strategy to overcome OS chemoresistance (125). Unfortunately, the above studies are relatively basic, if we can use electron microscopy and other experimental means to observe the autophagy behavior of osteosarcoma cells wound greatly increase the significance of the experimental results. Since the effect of autophagy on tumors is bidirectional, the different activation degree of PI3K/AKT pathway will lead to different degrees of autophagy activation, which may eventually produce tumor suppressor or carcinogenic effects. Therefore, it is not only a challenge but also an opportunity to study the relationship between PI3K/AKT and tumor autophagy.

#### 2.2.5 EMT

**Figure 4:**
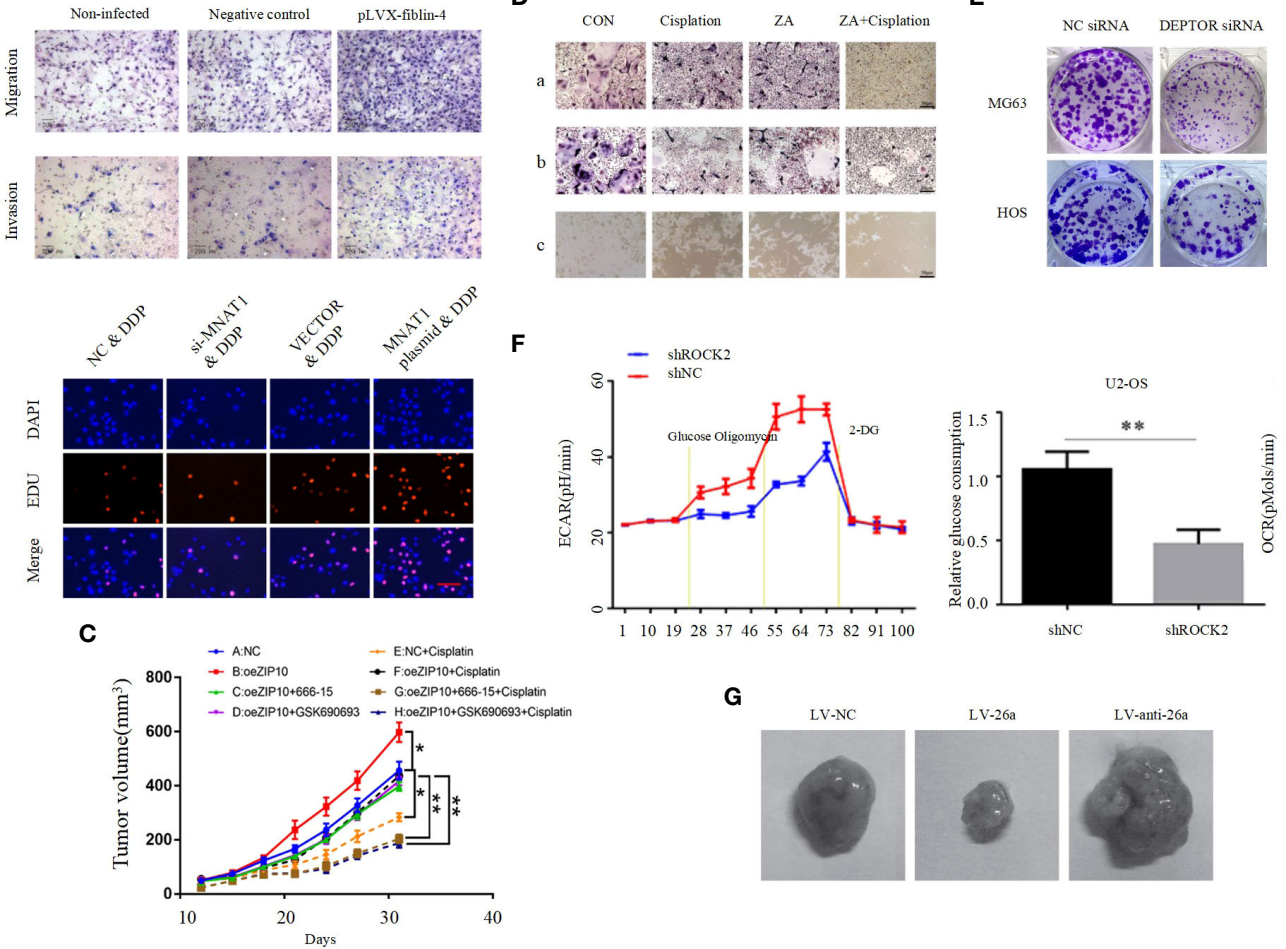
**(A)** Effect of fibulin-4 knockdown and overexpression on the migration and invasion of the differently invasive osteosarcoma cell subclones (135). **(B)** MNAT1 regulated OS chemo-sensitivity to DDP-based therapy (136). **(C)** Tumor weight in each group (54). **(D)** Cisplatin in synergy with ZA inhibited osteoclast formation, survival, and activation. **(a)** The TRAP staining of BMMs treated with M-CSF and RANKL for 4 days in the presence of cisplatin/ZA+cisplatin. **(b)** The mature osteoclasts were treated by cisplatin/ZA+cisplatin. **(c)** The bone resorption on Corning Osteo Assay 24-well plates of osteoclasts treated by cisplatin/ZA+cisplatin (65). **(E)** Colony formation capacity of osteosarcoma cells (97). **(F)** ROCK2 affects the level of glycolysis in OS cells. Extracellular acidification rate data revealed the glycolytic rate and capacity (137). **(G)** Tumor growth in mouse xenograft models. MG-63 cells infected with NC, miR-26a, or anti-miR-26a lentivirus were injected subcutaneously into nude mice (138). *p < 0.05, **p < 0.01.

EMT is a process of cell morphological characteristics change, which is characterized by the loss of epithelial cell phenotype and the transition to mesenchymal cell phenotype. The main manifestations include the down-regulation of epithelial cell markers, unstable cell-cell junctions, loss of basement membrane and apical polarity, and reorganization of the cytoskeleton (133). Although more attention has been paid to epithelial cancers, EMT also plays an important role in the formation of non-epithelial cancers, such as OS. EMT in OS is highly complex and regulated by multiple signaling pathways, including the PI3K/AKT pathway. E-calmodulin is a marker of primary epithelial tumors, and Shenyu Wang et al. found that by affecting the PI3K/AKT pathway can affect E-calmodulin levels and N-calmodulin levels in osteosarcoma cells, further altering the cell viability and EMT of osteosarcoma cells (**Figure 3C**) (126). Absent in melanoma 2 (AIM2) and Fer-1-like protein 4 (FER1L4) are two osteosarcoma inhibitors, and several studies have found that their overexpression inhibited the PI3K/AKT/mTOR signaling pathway, with an increase in E-calmodulin and a decrease in wave proteins and fibronectin, inhibiting EMT (**Figure 3D**) (61, 134). In contrast, STEAP2 (sixtransmembrane epithelial antigen of prostate 2), zinc finger CCHC domain containing 12 gene (ZCCHC12) (**Figure 3E**), and fibulin-4 (**Figure 4A**) are promoters of osteosarcoma, which can be induced by the PI3K/AKT/mTOR pathway to induce EMT and promote tumor growth, invasion and metastasis of osteosarcoma cells (104, 135, 139).

#### 2.2.6 Resistance to chemotherapy drugs

It is self-evident that chemotherapy plays an important role in the treatment of osteosarcoma. The application of chemotherapy has greatly improved the overall survival rate and quality of life of OS patients (140). However, the problem of chemoresistance has become increasingly prominent in recent years. The problem of chemoresistance has become a major obstacle to further improve the treatment effect of osteosarcoma patients. Chemotherapy resistance in OS can be mediated by a variety of mechanisms, mainly including significantly reduced intracellular drug accumulation, accelerated drug inactivation, increased DNA repair rate, disturbance of intracellular signal transduction pathways, and abnormal changes in apoptosis, autophagy and CSCs (124, 125, 141). The PI3K/AKT pathway affects chemoresistance in osteosarcoma has been demonstrated in several studies. Menage a trois 1 (MAT1) is a subunit in the cell cycle protein-dependent kinase-activated kinase (CAK) complex, and Chensheng Qiu et al. demonstrated that MAT1 is required to regulate OS chemosensitivity to DDP and achieves its action through the PI3K/AKT/mTOR pathway (**Figure 4B**) (136). A recent study found that knockdown of Zrt and Irt-related protein 10 (ZIP10) inhibited OS cell proliferation and chemoresistance, and that ZIP10 promoted Zn content-induced phosphorylation and activation of cAMP response element binding protein (CREB), which is a key component of integrin α10 (ITGA10) transcription and ITGA10 activation PI3K/AKT pathway, and does not stimulate the classical FAK or SRC pathways. It was further confirmed by *in vivo* experiments that ZIP10 mediates chemotherapy resistance in OS cells *via* the ITGA10-PI3K/AKT axis (**Figure 4C**) (54). Zoledronic acid (ZA) is a diphosphate compound used to treat bone diseases. It inhibits bone destruction caused by increased osteoclast activity (142). Previous studies found ZA to inhibit a variety of tumors including osteosarcoma, cervical and breast cancers (143–145). A recent study by Liang Liu et al. found that ZA combined with cisplatin significantly inhibited the malignant biological behavior of 143B cells, and that agonists of the PI3K/AKT pathway could reverse this result. This shows that ZA enhances the antitumor effects of cisplatin in osteosarcoma through the PI3K/AKT pathway and reduces chemoresistance and osteoclast activation (**Figure 4D**), and this study raises the possibility of using ZA in combination with cisplatin as a new strategy representing the fight against osteosarcoma (65). However, these studies related to drug resistance were supported only by cell and animal experiments without clinical trials. For example, YU et al. found that AMD 3100 could enhance the antitumor effect of adriamycin in an *in situ* OS mouse model. Decreased expression of p-PI3K, p-AKT, and p-mTOR were also observed. These results suggest that AMD 3100 promotes the antitumor effect of adriamycin on tumor growth *in vivo* (125).

#### 2.2.7 Angiogenesis

Angiogenesis is not only essential for normal life activities, but is also crucial for tumors, as tumor growth also requires blood vessels to provide them with adequate nutrients. One study reported that Nude mice injected with human OS 3AB-OS pluripotent CSC showed high AKT levels along with a significant increase in tumor vascular density, and the significant increase in vascular density was suppressed after inhibition of the PI3K/AKT pathway (146). This experiment suggests that activation of the PI3K/AKT pathway is important for angiogenesis in OS. Some studies found that TGF, VEGF, PDGF and basic fibroblast growth factor (bFGF) play a pro-angiogenic role in OS progression (147, 148). As mentioned above, as activators, they can effectively activate the PI3K/AKT pathway. Thus, activated PI3K/AKT pathway plays a key role in TGF, PDGF and bFGF-induced OS angiogenesis, however, its clear mechanism still needs further in-depth study. Recently, DEP domain-containing mTOR interacting protein (DEPTOR) has been identified as an endogenous mTOR inhibitor. Considering the close relationship between DEPTOR and mTOR, DEPTOR is thought to play an important role in the pathogenesis of many cancers, and it was found that DEPTOR overexpression significantly inhibits mTOR and activates the PI3K/AKT pathway, which is required for OS cell proliferation, migration, invasion, angiogenic mimetic formation and survival (**Figure 4E**) (97).

#### 2.2.8 Aerobic glycolysis

Increased aerobic glycolysis (Warburg effect) has become a hallmark of becoming cancerogenesis that can provide more intermediates for certain biosynthetic pathways and adaptation to hypoxic environments, a metabolic shift that leads to cancer cell proliferation and survival (149, 150). Rho-associated coiled-coiled-coil containing protein kinase 2 (ROCK2) is a serine-threonine kinase. As a downstream effector of the Rho subfamily of small gtpase, ROCK2 regulates cell morphology and migration. In osteosarcoma cells, ROCK2 can promote OS cell growth by inducing aerobic glycolysis. ROCK2 induces aerobic glycolysis mainly by activating p-PI3K/AKT pathway to promote the expression of mitochondrial hexokinase II (HKII). (**Figure 4F**) (137). Glucose metabolism assays demonstrated that PDGF/PDGFR-β effectively promoted aerobic glycolysis in osteosarcoma cells. PI3K/AKT pathway inhibitor LY294002 was used to perform WB assays and glucose metabolism assays. The results showed that PDGF/PDGFR-β promoted aerobic glycolysis in osteosarcoma cells mainly through the activation of PI3K/AKT/mTOR/c-Myc pathway (151).

## 3 MicroRNAs in the PI3K/AKT pathway

**Figure 5:**
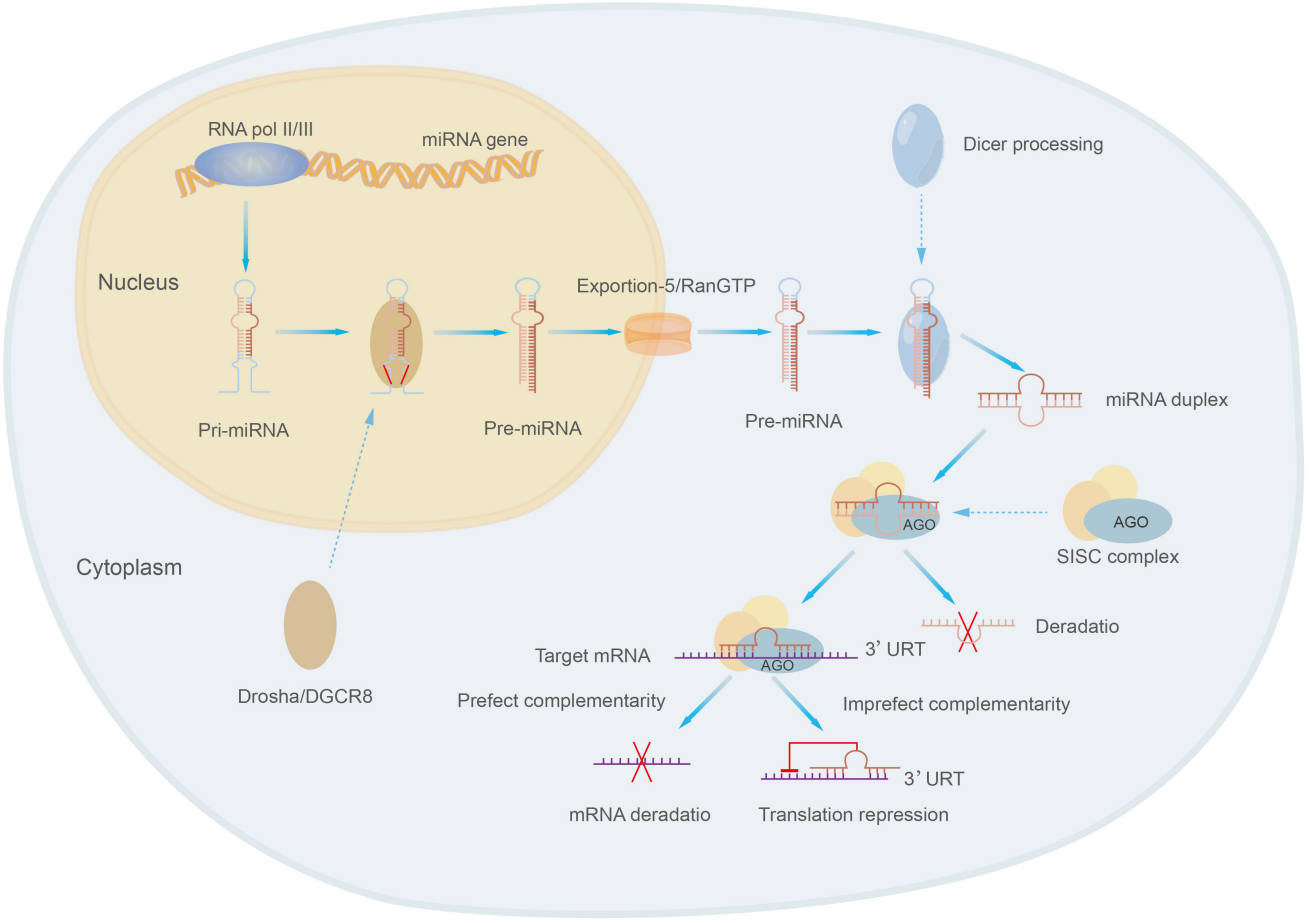
The formation process of miRNA.

MicroRNAs are class of small molecular RNAs of 21-25 nucleotides in length, they are widely found in animals, plants and eukaryotic microorganisms and are a novel and important factor in regulating gene expression (19, 152). MicroRNAs were originally identified in Cryptobacterium hidradenum (153). MicroRNAs exist in several forms, including pri-miRNA, pre-miRNA and miRNA. Pri-miRNA is derived from the genome of eukaryotes and is transcribed and spliced to form pre-miRNA, then pre-miRNA is sheared into mature double-stranded microRNA by the nucleic acid endonuclease Dicer in the extracellular nucleus, and the mature double-stranded microRNA is associated with Argonaute (AGO) and forms a RNA-induced silencing complex (RISC) called the rna-induced silencing complex, which selects one strand of the double-stranded body to become a mature miRNA and discards the other strand (152, 154) (**Figure 5**). MiRNA present a single-stranded form structurally and, most characteristically, contain a structurally stable base pair between the RNA and the RNA within the precursor RNA structure. The expression of miRNA tends to be higher due to the wide intracellular distribution of small RNAs (155).

MicroRNAs mainly regulate gene expression by targeting with specific proteins in cooperation with specific mechanisms: miRNAs select appropriate mRNA targets through the interaction of the initiating RNA-mediated silencing complex (RISC) with the mRNA 3’-UTR complementary sequences of target genes, target recognition, physical hindrance of target sequences, translation inhibition, degradation and other steps to affect the expression of target genes and their functions, thus participating in the regulation of various cell biological processes, such as cell proliferation, apoptosis, differentiation and metabolism (106, 156, 157). For example, the interaction of miR-181a-5p with the 3’-UTR complementary sequence of PTEN leads to a decrease in PTEN, which results in the activation of PI3K/AKT pathway further affecting the development of osteosarcoma (55).

PI3K/AKT pathway is the main signaling pathway that affects the biological behavior of tumor cells. There is a close relationship between miRNA and PI3K/AKT pathway. On the one hand, miRNA can affect the activation and inhibition of the signaling pathway by targeting and regulating the expression of PI3K/AKT pathway-related genes. For example, in osteosarcoma cells miR-181a-5p (55) and miR-133a (158) can target PTEN genes and inhibit their expression, thus promoting the activation of PI3K/AKT pathway. In contrast, microRNAs such as miR-506-3p (159) and miR-126 (160) can target genes of the PI3K/AKT pathway and suppress their expression, thus inhibiting the activation of the signaling pathway. On the other hand, the PI3K/AKT pathway can also function by regulating the expression of miRNA. For example, the activation and inhibition of AKT can also affect the expression of miR-21, which further affects the activation of PI3K/AKT pathway (161).

In summary, miRNA and PI3K/AKT pathway are interacting with each other, they regulate each other, and for the occurrence and development of some diseases, such as tumorigenesis and metastasis, they are also related to the abnormalities of miRNA and PI3K/AKT pathway. Therefore, studying the interaction between miRNA and PI3K/AKT pathway in osteosarcoma is of great significance for further systematic and in-depth understanding of the pathogenesis of part of osteosarcoma and the development of new and effective diagnostic, therapeutic and prognostic strategies.

## 4 Role of microRNA/PI3K/AKT axis in osteosarcoma

Many microRNAs associated with the PI3K/AKT pathway are aberrantly expressed in osteosarcoma. A new series of studies shows that a range of clinical features in patients with osteosarcoma are associated with the PI3K/AKT pathway and microRNAs (**Table 1**). In addition, the microRNA/PI3K/AKT axis promotes cancer progression by altering various biological properties of osteosarcoma cells. In this article, we will introduce in detail the Status of expression, clinical features, basic functions, and mechanism of microRNA/PI3K/AKT axis from two parts: cancer suppressor factors (**Table 2**) and cancer promoting factors (**Table 3**) that act on the PI3K/AKT pathway. It provides certain directions and ideas for further insight into the mechanisms associated with microRNA/PI3K/AKT axis-related osteosarcoma and the development of new therapeutic strategies.

**Table 1:** Clinical characterization of miRNAs associated with the PI3K/AKT pathway.

MicroRNA	Expression	Targeted	Object	Prognostic indicator	Clinical feature	Ref
miR-3927-3p	Downregulated	Integrin-αvβ3	7 patients undergoing surgical resection		Clinical stages,lung metastasis	(162)
miR-106	Upregulated	PI3K	54 tumor tissues and adjacent normal tissues (35 males and 18 females with the mean age of 12.3 years, including 32 cases with lung metastasis)		Lung metastasis, clinical stages	(163)
miR-183-5p	Downregulated	AKT	40 OS patients were enrolled in this study, including 24 males and 16 females (6-48years, mean age of 26.24 ± 6.58 years)	Overall survival	Distant metastasis	(164)
miR-130a	Upregulated	PTEN	Tumor specimens and the adjacent non-cancer tissues were obtained from 86 osteosarcoma patients	Overall survival, disease-free survival	Clinicopathologic feature,TNM staging,Distant metastasis	(56)
miR-340-5p	Downregulated	NRF2	47 tumor tissues and adjacent normal tissues	Overall survival	Malignant clinicopathologic features	(165)
miR-21	Upregulated	PTEN,tgf-β1	46 patients with osteosarcoma(26 males and 20 females, and age ranged from 10 to 65 years, with an average age of 32 ± 9.2 years)		TNM staging,Late diagnosis	(166)
miR-133b	Downregulated	FGFR1	30 OS patients(15 males and 15 females)		Malignant lesion, advanced clinical stage,distant metastasis	(167)
miR-95-3p	Upregulated	TGF-β	Peripheral blood of OS patients and normal volunteers		Clinical stage	(168)
miR-506-3p	Downregulated	RAB3D	30 tumor tissues and adjacent normal(21 of the patients were male,The average age of the patients was 21.21 years (range: 11 to 41 years))	Overall survival		(159)
miR-200c	Upregulated	pAKT	36 cases of primary osteosarcoma	Overall survival		(169)
miR-92a	Upregulated	PTEN	68 OS and 20 normal bone tissue samples were obtained	Event-free survival,overall survival	Clinical stage, T classification,histological differentiation	(170)
miR-615	Downregulated	HK2	92 paired OS tissues were collected from OS patients (60 males and 32 females, mean age 21 years, range from 14 to 33 years)	Overall survival	TNM stage,lymph node metastasis	(59)
miR-1908	Upregulated	PTEN	46 paraffin-embedded osteosarcoma specimens and 9 normal muscle samples	Overall survival	Clinical stage	(60)
miR-148a	Upregulated	PTEN	Human osteosarcoma tumor tissues and paired adjacent normal tissues were obtained from 92 primary osteosarcoma	Overall survival	Clinical stage	(171)
miR-449a	Downregulated	EZH2	48 OS and adjacent normal tissue specimens were collected by excision biopsy from OS patients	Overall survival	Malignant clinicopathologic features,	(172)
miR-373	Upregulated	p53	22 paired Primary spinal OS and matched normal non-tumor tissues		TNM stage,tumor size	(173)
miR-141-3p	Downregulated	EGFR	Tumor tissues and adjacent normal tissues were obtained from 32 patients	Overall survival	TNM stage	(174)
miR-499a-5p	Downregulated	PPM1D	62 primary OS and adjacent Non-tumorous tissue samples(ratio of male and female was 37: 25 and the average age was 19.3 ± 4.5 years.)		TNM stage,clinical stage	(175)
miR-532-5p	Upregulated	PTEN	50 osteosarcoma tissue specimens and paired paratumor tissue specimens	Overall survival	Clinical stage,Distant metastasis, tumor size,Lymph node metastasis	(176)
miR-128	Upregulated	PTEN	100 pairs of osteosarcoma tissue samples(The patients ranged from 8 to 68 years of age (median 18 years, mean 22.68 years). The study population was 68 males and 32 females)	Overall survival,disease-free survival	Adverse reactions to chemotherapy,positive metastases	(177)
miR-29c -3p	Downregulated	TPX2	52 pairs of primary osteosarcoma and adjacent noncancerous tissues(age range, 2-54 years; average age,19.47 ± 3.30 years)	Overall survival	Tumor size, clinical stage,distant metastasis	(178)
miR-520c-3p	Downregulated	AKT-1,P65	29 OS and 8 normal tissue samples (contain-ing 8 pairs of samples)		Tumor metastasis,clinical stage	(179)
miR-149-5p	Downregulated	Fn14	The clinicopathological and prognostic data for 191 sarcoma patients and 66 adjacent normal samples were downloaded from The Cancer Genome Atlas 2015 RNA sequencing database	Overall survival	Tumor size,	(180)
miR-195-5p	Downregulated	FGF2	55 osteosarcoma tissue samples and corre-sponding adjacent non-tumor tissues	Overall survival	Tumor size,clinical stage,Distant metastasis	(181)
miR-214	Upregulated	PHLDA2	Tumor tissues and paired non-tumor tissues were obtained from 30 patients		Lung Metastasis	(182)

**Table 2:** Cancer suppressor acting on the PI3K/AKT pathway.

microRNA	Role	Targeted	Functions	Animal/Human	Cell line	Refs
miR-3927-3p	Tumor suppressor	Integrin-αvβ3	Proliferation, migration, invasiveness		MG-63, HOS,143B	(162)
miR-133a	Tumor suppressor	IGF1R	proliferation, invasion and metastasis	Nude mouse	Saos-2, HOS, U-2OS,hFOB	(158)
miR-134	Tumor suppressor	VEGF,VEGFR1	Proliferation, Angiogenesis	Nude mouse,11 OS tissues and 6 non-tumor bone tissues	MG-63, U-2OS,Saos-2	(183)
miR-183-5p	Tumor suppressor	AKT	Proliferation, metastasis, migration, colony formation, cell viability	40 tumor tissues and adjacent normal tissues	Saos-2,MG63,hFOB	(164)
miR-340-5p	Tumor suppressor	NRF2	EMT, Proliferation, Metastasis, migration, invasion, cell viability	Tumor tissues and adjacent normal tissues	U-2OS,Saos-2,MG-63,HOS,hFOB 1.19	(165)
miR-124	Tumor suppressor	TGF-β	Proliferation, migration, invasion,apoptosis, Colony formation	Nude mice	MG-63,MC3T3 -E1	(57)
miR-26a	Tumor suppressor	IGF-1	Proliferation	Nude mice,32 tumor tissues and adjacent normal	U-2OS,MG-63	(184)
miR-133b	Tumor suppressor	FGFR1	EMT, Proliferation, migration, invasion, apoptosis	30 tumor tissues and adjacent normal	hFOB1.19,U-2OS,MG-63,Saos-2	(167)
miR-506-3p	Tumor suppressor	RAB3D	Proliferation, metastasis, invasion	Nude mice,30 tumor tissues and adjacent normal	hFOB 1.19, HOS, U-2OS,MG-63	(159)
miR-22	Tumor suppressor	PI3K	Chemoresistance continues, autophagy,proliferation	Nude mice	MG63,U-2OS,Saos-2,OS9901	(185)
miR-532-3p	Tumor suppressor	AKT3	Proliferation, migration, invasion	Nude mice,30 patients	hFOB 1.19,MG63,U-2OS	(186)
miR-29b	Tumor suppressor	Spin 1	Proliferation, migration		U-2OS,GM-63	(187)
miR-615	Tumor suppressor	HK2	EMT, Proliferati, Metastasis, cell viability	92 paired OS tissues were collected from OS patients	hFOB1.19,HOS	(59)
miR-223	Tumor suppressor	Ect2	Proliferati, Cell cycle	15 pairs of Osteosarcoma and adjacent noncancerous tissue	MG-63, Saos-2, U-2OS	(188)
miR-206	Tumor suppressor	PAX3,MET	Proliferation, metastasis, migration, invasion, apoptosis	25 pairs of Osteosarcoma and adjacent noncancerous tissue	OS primary cell	(189)
miR-375	Tumor suppressor	mTOR	Proliferation, apoptosis, autophagy	20 pairs of osteosarcoma and adjacent noncancerous tissue	MG-63, HOS, U-2OS,Saos-2,hFOB1.19	(190)
miR-101	Tumor suppressor	ROCK1	Proliferation, Migration, invasion, apoptosis	Human osteosarcoma tissues and the corresponding normal osteoblast (n = 20)	MG-63,U-2OS,OS732	(191)
miR-18a	Tumor suppressor	MED27	Migration, invasion, cell viability, apoptosis		MG-63,Saos-2	(192)
miR-122	Tumor suppressor	TP53	Proliferation, cell cycle	50 pairs of osteosarcoma and adjacent noncancerous tissue	U-2OS,MG-63	(193)
miR-100	Tumor suppressor	IGFIR	Proliferation, migration, invasion, chemical resistance	20 pairs of osteosarcoma and adjacent noncancerous tissue	HOS, U-2OS, Saos-2, MG-63	(194)
miR-133b	Tumor suppressor	AKT	Proliferation, migration, Cell viability, apoptosis		MG-63	(195)
miR-144	Tumor suppressor	mTOR	Proliferation, apoptosis		MG-63,u-2 OS	(196)
miR-451a	Tumor suppressor	YTHDC1	EMT, Proliferation, invasion, apoptosis, colony formation		hFOB1.19,HOS,MG-63,U-2OS,143B	(26)
miR-449a	Tumor suppressor	EZH2	EMT, Proliferation, invasion, migration	48 OS and adjacent normal tissue specimens were collected by excision biopsy from OS patients	hFOB1.19,U-2OS,Saos-2,MG-63, HOS	(172)
miR-141-3p	Tumor suppressor	EGFR	Proliferation, migration, cell growth, apoptosis	Tumor tissues and adjacent normal tissues were obtained from 32 patients	hFOB1.19,HOS,MG-63	(174)
miR-139	Tumor suppressor	ROCK1	Proliferation, migration, invasion	Nude mice,25 paired OS and 19 non-tumor tissue samples	HOS, Saos-2, MG-63, U-2OS, OS732,hFOB1.19	(197)
miR-133b	Tumor suppressor	IGF1R	Proliferation, migration, invasion, apoptosis	23 pairs of osteosarcoma and adjacent noncancerous tissue	U2-OS,MG-63	(198)
miR-142	Tumor suppressor	CDK6	Proliferation, migration, invasion, apoptosis	Nude mice,45 paired OS tissues and matched non-tumorous tissues,	hFOB1.19,MG-63,SOSP-9607,HOS,U-2OS,Saos-2	(199)
miR-499a-5p	Tumor suppressor	PPM1D	Proliferation	62 primary OS and adjacent non−tumorous tissue samples	MG-63	(175)
miR-497	Tumor suppressor	VEGFA	Proliferation, Drug resistance	14 primary osteosarcoma and corresponding noncancerous tissue	Saos-2	(200)
miR-375	Tumor suppressor	PIK3CA	Proliferation	30 paired OS and matched normal non-tumor tissues	HOS, Saos-2, U-2OS, and MG-63,hFOB1.19	(201)
miR-6888-3p	Tumor suppressor	SYK	Proliferation, migration, cell viability	Nude mice,32 pairs of OS tissues and adjacent nor­ mal bone were obtained from patients	HOS,Saos-2,MG-63,SW1353,HFOB1.19	(202)
miR-384	Tumor suppressor	SLBP	Metastasis, migration, invasion, apoptosis, cell viability	Human osteosarcoma tissues and the corresponding normal tissues	hFOB1.19,MG-63,U-2OS,OS732	(203)
miR-141,miR-146b-5p	Tumor suppressor	AUF1,PDK1	EMT, Proliferation, migration, invasion		EH1,Saos-2,U-2OS,HOS,143B	(204)
miR-223	Tumor suppressor	Hsp90B1	Proliferation, apoptosis, cell cycle,cell growth	Nude mice	MG-63	(205)
miR-410	Tumor suppressor	VEGF	Proliferation, migration, invasion, apoptosis, cell growth	Nude mice, OS specimens and paired adjacent specimens (n = 15)	Saos-2,MG-63	(206)
miR-29c -3p	Tumor suppressor	TPX2	Proliferation	52 pairs of primary osteosarcoma and adjacent noncancerous tissues	MG-63,U-2OS,hFOB	(178)
miR-520c-3p	Tumor suppressor	AKT-1,P65	Proliferation,metastasis	Nude mice,29 OS and 8 normal tissue samples	143B	(179)
miR-152	Tumor suppressor	c-MET	Proliferation, cell growth, apoptosis, drug resistance	Nude mice	MG-63	(207)
miR-340-5p	Tumor suppressor	PI3K	Drug resistance	Nude mice	mg63-cr,Saos-2-cr	(208)
miR-141-3p	Tumor suppressor	SIX1	Proliferation, migration, invasion, apoptosis	25 osteosarcoma tissue samples and corresponding adjacent non-tumor tissues	HOS,Saos-2,143B,U-2OS,MG63	(209)
miR-29a-3p	Tumor suppressor	IGF1	Proliferation, migration, invasion, apoptosis, autophagy, colony formation	The OS tissues and adjacent non-tumor tissues	143B,MG-63,HOS,SJSA-1,hFOB1.19	(210)
miR-122-5p	Tumor suppressor	TP53	Proliferation, apoptosis, cell cycle	50 cases of osteosarcoma tissue samples	Hs888T,U-2OS,MG-63	(193)
miR-149-5p	Tumor suppressor	Fn14	Proliferation, cell growth, colony formation		U-2OS,Saos-2,MG-63,SW-1353,HOS,143B	(180)
miR-485-5p	Tumor suppressor	AKT1,HSP90	Proliferation, migration	20 paired adjacent normal tissues	HOB,C-12720,U-2OS	(211)
miR-652	Tumor suppressor	HOXA9	Proliferation, migration, invasion	30 osteosarcoma tissue samples obtained from osteosarcoma surgery	hFOB1.19,HOS,U-2OS,SJSA1,Saos-2,MG-63	(212)
miR-1224-5p	Tumor suppressor	PLK1	Apoptosis, autophagy, proliferation, invasion		hFOB1.19,MG-63,U-2OS,Saos-2,HOS,143B,	(213)
miR-195-5p	Tumor suppressor	FGF2	Apoptosis, metastasis, proliferation,	Nude mice,55 osteosarcoma tissue samples and corre- sponding adjacent non−tumor tissues	hFOB1.19,143B,U-2OS,MG-63,MNNG,Saos-2	(181)

**Table 3:** Oncogenic factors acting on PI3K/AKT pathway.

microRNA	Role	Targeted	Functions	Animal/Human	Cell line	Ref
miR-181a-5p	Oncogene	PTEN	Proliferation, colony formation, migration, invasion, and cell cycle progression of osteosarcoma cells,Chemotherapy drug sensitivity		Saos-2, MG-63, HOS, 143B,U-2OS,hFOB1.19	(55)
miR-19a-3p	Oncogene	PTEN	Osteoclast differentiation and bone destruction through	Mouse	K7M2, MG-63, HOS, RAW264.7	(214)
miR-216	Oncogene	PTEN	Cell proliferation, cell cycle, cell invasion,migration, cell apoptosis	Tumor tissues and adjacent normal tissues(n=10)	MG-63	(215)
miR-208a-3p	Oncogene	PTEN	Proliferation, migration, and invasiveness	OS tissue specimens (n=10) and adjacent normal tissues	Saos-2, U-2OS,MG-63	(216)
miR-214	Oncogene	PTEN	invasion, metastasis, Drug sensitivity, tumor volume growth, apoptosis	Nude mouse	U-2OS,MG-63	(58)
miR-524	Oncogene	PTEN	Proliferation,apoptosis		MG-63, 143B, Saos-2 and UMR-106,hFOB 1.19	(217)
miR-9-5p	Oncogene	PTEN	Proliferation, Cell viability, apoptosis	17 tumor tissues and adjacent normal tissues	MG-63,U-2OS, 143B,hFOB1.19	(218)
miR-106	Oncogene	PI3K	Proliferation, cell cycle, invasion, metastasis, cell cycle	54 tumor tissues and adjacent normal tissues	U2-OS	(163)
miR-620	Oncogene	PTEN	Proliferation, migration, apoptosis	Nude mice	MG-63,KHOS,hFOB1.19	(219)
miR-130a	Oncogene	PTEN	EMT, Metastasis, migration, invasion	86 tumor tissues and adjacent normal tissues	HOS58,Saos-2,MG-63	(56)
miR-17	Oncogene	SASH1	Proliferation, migration, invasion, apoptosis		U-2OS,MG-63	(220)
miR-21	Oncogene	PTEN,tgf-β1	Proliferation	46 tumor tissues and adjacent normal tissues	U-2OS,MG-63	(166)
miR-23b-3p	Oncogene	VEPH1	Proliferation, migration, cell viability	24 tumor tissues and adjacent normal	HOS,hFOB1.19,SJSA-1, Saos-2, U-2OS	(184)
miR-199a-5p	Oncogene	PIAS3,p27	Proliferation and tumour growth	Nude mice,8 tumor tissues and adjacent normal	Saos-2,MNNG,HOS	(221)
miR-95-3p	Oncogene	TGF-β	Cell growth, apoptosis	Peripheral blood of OS patients and normal volunteers	F5M2	(168)
miR-196a	Oncogene	PTEN	Proliferation,apoptosis,cell cycle		MG-63,HOS,Saos-2,U-2OS,hFOB1. 19	(222)
miR-21	Oncogene	PTEN	Proliferation, metastasis, invasion		MG-63, hFOB1.19	(223)
miR-221	Oncogene	PTEN	Proliferation, cell cycle, Cell viability, apoptosis, Drug sensitivity	36 primary and 24 recurrent human osteosarcoma tissues and 25 normal adjacent tissues	MG63,Saos-2,U-2OS,hFOB1.19	(94)
miR-214	Oncogene	PTEN	Proliferation, apoptosis	Nude mice,15 human primary OS and matched adjacent noncancerous tissues	Saos-2,hFOB1.19	(224)
miR-802	Oncogene	P27	Migration, intrusion, and EMT	68 paired of OS tissues and adjacent non- OS tissues	143B, HOS, MG-63, U-2OS,Saos- 2,hFOB	(225)
miR-92a	Oncogene	PTEN	Proliferation, cell cycle, apoptosis	Nude mice,68 OS and 20 normal bone tissue samples were obtained	MG-63,U-2OS	(170)
miR-214-3p	Oncogene	PTEN	Proliferati, cell viability, apoptosis		MG-63	(226)
miR-93	Oncogene	PTEN	Proliferati, cell viability, apoptosis, Cell cycle	Nude mice	HOS,Saos-2,MG-63,NY,Hu09,hMSCs	(227)
miR-1908	Oncogene	PTEN	Proliferation, invasion, colony formation	Nude mice,46 paraffinembedded osteosarcoma specimens and 9 normal muscle samples	143B,U-2 OS,MG-63,Saos-2,hFOB 1.19	(60)
miR-21	Oncogene	PTEN	Proliferation, apoptosis		Saos-2,MG- 63	(228)
miR-17	Oncogene	PTEN	Proliferation, Migration, invasion,	28 pairs of osteosarcoma and adjacent noncancerous tissue	U-2OS,Saos-2,MG-63	(95)
miR-181a	Oncogene	PTEN	Cell viability, apoptosis, invasion	Primary OS tissues and the adjacent non-tumor tissues were obtained from 20 patients treated	Saos-2,U-2OS	(229)
miR-148a	Oncogene	PTEN	Cell growth	92 pairs of OS and their matched adjacent normal tissues	hFOB1.19,MG-63,U-2OS	(171)
miR-181b	Oncogene	PTEN	Proliferation	22 pairs of OS and their matched adjacent normal tissues	Saos-2, U-2OS, MG63,hFOB	(230)
miR-21	Oncogene	PTEN	Proliferation, invasion, Cell viability,		MG-63,saos-2	(231)
miR-214	Oncogene	PTEN	Proliferation, migration, invasion, cell viability	22 paired OS and matched normal non-tumor tissues	U-2OS,saos-2,mg-63	(232)
miR-373	Oncogene	p53	Proliferation, migration, invasion, cell viability, colony formation	22 paired Primary spinal OS and matched normal non-tumor tissues	hFOB1.19,Saos-2,MG-63,U-2OS	(173)
miR-18a-5p	Oncogene	SOCS5	EMT, Proliferation, apoptosis	25 paired OS tissues and matched non-tumorous tissues	hFOB1.19,MG-63,U-2OS,HOS,Saos-2	(233)
miR-532-5p	Oncogene	PTEN	Proliferation, migration	Nude mice,50 osteosarcoma tissue specimens and paired paratumor tissue specimens	hFOB 1.19,MG-63, U-2OS, MNNG/HOS, 143B,	(176)
miR-25	Oncogene	p27	Proliferation	Nude mice,25 osteosarcoma tissue specimens and paired paratumor tissue specimens	Saos-2,U-2OS	(234)
miR-128	Oncogene	pten	Invasion	100 paired OS tissues and matched non-tumorous tissues		(177)
miR-106b-5p	Oncogene	P27	Proliferation, migration, cell cycle	18 pairs of fresh surgically resected OS tissue and adjacent bone tissue	Saos-2,MG-63, SW1353,U-2OS, hFOB 1.19	(235)
miR-21	Oncogene	PTEN	Proliferation, invasion and apoptosis		MG-63,hFOB1.19	(236)
miR-744	Oncogene	PTEN	Proliferation	25 osteosarcoma tissue samples and corre- sponding adjacent non-tumor tissues	MG-63,U-2OS,Saos-2,hFOB1.19	(237)
miR-214	Oncogene	PHLDA2	Apoptosis, radiosensitivity	Nude mice,Tumor tissues and paired non-tumor tissues were obtained from 30 patients	HEK293,MG-63,U-2OS,HOS,Saos-2	(182)

### 4.1 Cancer suppressor acting on the PI3K/AKT pathway

**Figure 6:**
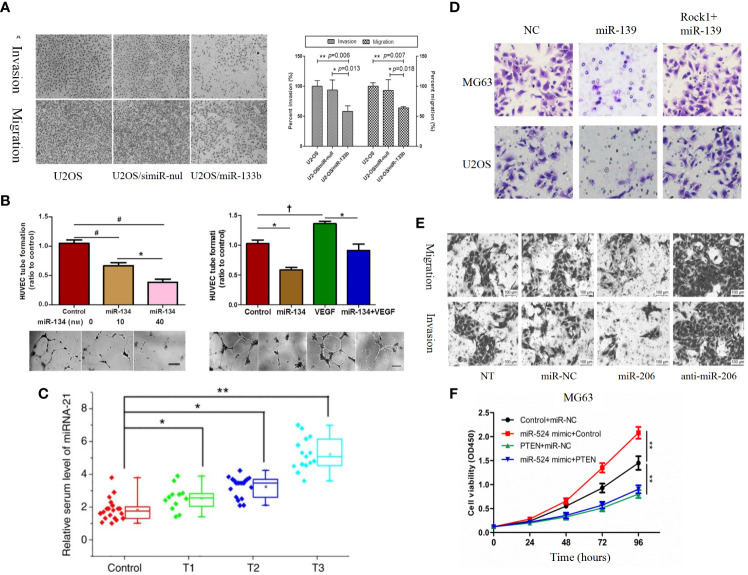
**(A)** Roles of miR-133b in invasion and migration of osteosarcoma cell lines. Over-expression of miR-133b suppressed cell invasion and migration in U2-OS cells. Cell invasion and migration were visualized and examined by inverted phase contrast microscope (198). **(B)** MiR-134 overexpression inhibits the proliferation of Saos-2 cells and their secretion of angiogenesis factors *in vitro*, but promotes Saos-2 cell apoptosis (183). **(C)** Serum levels of miR-21 increase as the T stage of osteosarcoma increases (166). **(D)** ROCK1 rescues cell proliferation and invasion in miR-139 overexpression OS cells. Representative images of transwell invasion assay (197). **(E)** Representative results of cell migration and invasion assays (189). **(F)** MG-63 **(a)** and 143 **(b)** cells are transfected with Control + miR-NC, miR-524 mimic + Control, PTEN + miR-NC or miR-524 mimic + PTEN, and cell proliferation in each group is detected by CCK-8 (217). ^†^P < 0.05; *P < 0.01; **P < 0.001, ^#^P < 0.0001.

Insulin-like growth factor-1 (IGF-1) is composed of 70 amino acids. IGF-1 can affect the proliferation, invasion, metastasis and drug resistance of tumor cells (238, 239). Recent studies found that miR-26a (**Figure 4G**) and miR-29a-3p were lowly expressed in osteosarcoma and negatively correlated with IGF-1 expression, and further studies revealed that overexpression of miR-26a and miR-29a-3p could target IGF-1 to inhibit the IGF-1R/PI3K/AKT pathway to affect apoptosis and autophagy in OS cells, as evidenced by a significant attenuation of invasion (138, 210). IGF-1 can act by activating the PI3K/AKT pathway through activation of the IGF-1R located on the cell surface, and therefore by affecting IGF-1R can also affect activation of the PI3K/AKT pathway.It was found that miR-133a, miR-133b (**Figure 6A**), and miR-100 can partially block this pathway, and miR-133a, miR-133b, and miR-100 can then target IGF-1R in human osteosarcoma cells to inhibit the activation of PI3K/AKT pathway and thus inhibit apoptosis, proliferation, invasion, metastasis, migration, and chemoresistance of osteosarcoma (158, 194, 198). VEGF can bind to VEGFR1 to activate PI3K/AKT pathway thereby promoting tumor proliferation and angiogenesis (240). MiR-134, miR-497 and miR-410 were found to be lowly expressed in osteosarcoma cells, and functionally microRNA-134, miR-497 and miR-410 could lead to reduced expression of VEGF and VEGFR1, thus inhibiting the PI3K/AKT pathway leading to osteosarcoma angiogenesis, proliferation, migration, invasion, cell growth, and drug resistance (**Figure 6B**) (183, 200, 206). MiR-124 and miR-21 are lowly expressed in osteosarcoma, and overexpression can act on TGF-β to affect the activation of PI3K/AKT pathway. As a result, the malignant degree of osteosarcoma cells decreased significantly. in addition miRNA -21 is also strongly associated with TNM staging in osteosarcoma patients (**Figure 6C**) (57, 166). Bingsheng Yang et al. found that miR-195-5p expression was significantly reduced in OS and negatively correlated with fibroblast growth factor-2 (FGF-2) expression. MiR-195-5p/FGF2/PI3K/AKT axis can affect the occurrence and metastasis of osteosarcoma (181). Fibroblast growth factor receptor-1 (FGFR-1) can mediate the activation of PI3K/AKT pathway. MiR-133b can target FGFR-1 in OS to inhibit PI3K/AKT pathway and thus inhibit the progression of osteosarcoma. MiR-195-5p and miR-133b were found by studying clinical samples 5p and miRNA-133b were also found to be closely associated with tumor size, distant metastasis and the clinical stage of the patients in OS patients, thus it is important to continue in-depth studies on miR-195-5p and miR-133b (167).

There are also many microRNAs whose effects on the PI3K/AKT pathway are directly on PI3K and AKT molecules, for example, miR-340-5p is under-expressed in osteosarcoma as an oncogenic factor, and thus as a direct target gene of miR-340-5p, PI3K receives significantly less direct inhibition, leading to abnormal PI3K/AKT pathway and a OS development with a significant contribution (208). MiR-22 significantly inhibits the proliferation of MG63 cell line and MG63/CDDP cells, and enhanced the anti-proliferation ability of CDDP *in vitro* and *in vivo*. In addition, miR-22 can also reduce CDDP-induced autophagy and mediate the resistance of osteosarcoma cell lines to CDDP (185). AKT has an extremely important position in the PI3K/AKT pathway (241).Several studies found that miR-183-5p, miR-532-3p, miR-133b, miR-520c-3p and miR-485-5p were overexpressed in osteosarcoma, and overexpression would directly affect the formation of AKT leading to abnormal PI3K/AKT pathway, and the proliferation, migration, cell viability, and apoptosis of osteosarcoma cells would be affected (164, 179, 186, 195, 211). In addition, miR-183-5p and miR-520c-3p are also associated with clinical characteristics and prognosis of OS patients, including distant metastasis of tumors and overall survival of patients at clinical stage. Therefore the interaction mechanism of miR-183-5p and miR-520c-3p with PI3K/AKT pathway should be investigated in depth (164, 179). PI3K and Akt molecules are the core of PI3K/Akt pathway and are the most important signaling molecules. Therefore, mirnas directly acting on PI3K and Akt molecules can more directly affect the abnormal activation of PI3K/Akt pathway, which may have a more significant effect on osteosarcoma cells, and reduce the influence of the whole criss-crossing signal network, which is worthy of our study.

Luciferase assay and bioinformatic analysis confirmed that mTOR is a direct target gene of miR-375-3p and miR-144. mTOR is a key regulator of various life activities of cells and performs its various functions mainly by participating in the PI3K/AKT pathway. Reduced expression of miR-144 miR-375-3p in osteosarcoma cells leads to PI3K/AKT/mTOR pathway aberrant activation, which affects OS cell proliferation, apoptosis and autophagy (190, 196). ROCK1 is a serine/threonine kinase belonging to the Rho family that promotes the reorganization of the actin cytoskeleton (242). It has been found that microRNA interaction with ROCK1 may be related to the formation and progression of osteosarcoma, where miR-101 and miR-139 have been demonstrated as osteosarcoma suppressors by targeting rock1. It was found that by overexpressing miR-101 and miR-139 (**Figure 6D**) can downregulate rock1 while causing PI3K/AKT and JAK/STAT signaling pathway inactivation, ultimately leading to the malignant behavior of osteosarcoma cells can be partially suppressed, including biological behaviors such as abnormal proliferation, migration and invasion (191, 197). MiR-340-5p (165), miR-506-3p (159), miR-615 (59), miR-18a (192), miR-122-5p (193), miR-451a (26), miR-449a (172), miR-142 (199), miR-499a-5p (175), miR-6888-3p (202), miR-384 (203), and miR-223 (205), miR-29c-3p (178), miR-152 (207), miR-149-5p (180), miR-652 (212), miR-1224-5p (213), miR-146b-5p (204), miR-141-3p (209), miR-141 [198], miR-29b (187), and miR-223 (188) are all lowly expressed in osteosarcoma cells as tumor suppressors, and they can act directly on NRF2, RAB3D, HK2, MED27, TP53, YTHDC1, EZH2, CDK6, PPM1D, SYK, SLBP, Hsp90B1, TPX2, c-MET, Fn14, HOXA9, PLK1, PDK1, SIX1, AUF1, Spin 1, Ect2 and thus inhibit the PI3K/AKT pathway, which ultimately leads to the impact of osteosarcoma cells in proliferation, migration, invasion, apoptosis, autophagy, cell cycle, cell growth, etc., and inhibits the development of osteosarcoma and progression of osteosarcoma. In addition, miR-206 can also act on both PAX3 and MET target genes to achieve the inhibition of PI3K/AKT pathway through two pathways, so the inhibitory effect of miR-206 on osteosarcoma may be more obvious (**Figure 6E**) (189). Systematic in-depth analysis of clinical data from multiple osteosarcoma patients found a significant correlation between the expression of miR-506-3p in osteosarcoma tissues and patient prognosis. Specifically, lower expression levels of miR-506-3p were associated with worse prognoses (159). And the expression of miR-499a-5p was significantly correlated with TNM staging, showing that the lower the expression of miR-499a-5p, the higher the grade of TNM staging (175). In contrast, low expression of miR-340-5p (165), miR-615 (59), miR-449a (172), miR-29c-3p (178), and miR-149-5p (180) not only predicted a poorer prognosis for patients, but also correlated with osteosarcoma TNM stage, clinical stage, tumor size, distant metastasis and other clinical features are significantly correlated. Therefore, this part of MicroRNA that has been shown to correlate with patient prognosis and clinical characteristics deserves further in-depth study and hopefully can be applied to clinical practice.

### 4.2 Oncogenic factors acting on PI3K/AKT pathway

PTEN is a member of the protein tyrosine phosphatase gene family and represents the first identified tumor suppressor with bispecific phosphatase activity to date. In the PI3K/AKT pathway, PTEN plays a crucial role in the regulation of cellular signaling pathways by dephosphorylating phosphatidylinositol-3,4,5-trisphosphate (PIP3), the second messenger produced by PI3K, thereby negatively modulating the activity of serine/threonine protein kinase AKT (243, 244). Thus PTEN has been shown to act as a tumor suppressor gene by inactivating the PI3K/AKT pathway (198), ultimately participating in the regulation of proliferation, cell cycle, apoptosis, migration, invasion and metastasis during cancer development (245). In osteosarcoma, a number of studies have shown that abnormalities in the PI3K/AKT pathway can be caused by the interaction of microRNA with PTEN, which is basically in a high expression state in osteosarcoma and is generally considered as a pro-oncogenic factor. For example, Chen Sun et al. found that miR-181a-5p, which is highly expressed in osteosarcoma cells, could bind to the 3-URT of PTEN and reduce its protein expression, thus activating the PI3K/AKT pathway. Overexpression of PTEN or inhibition of AKT significantly inhibited the tumor-promoting effect of miR-181a-5p (55). Ming Zhuang et al. found that miR-524 was significantly upregulated in osteosarcoma tissues and osteosarcoma cell lines, miR-524 knockdown inhibited proliferation and promoted apoptosis in osteosarcoma cells, and bioinformatics analysis and luciferase analysis confirmed that PTEN was a direct target gene of miR-524. miR-524 activated PPI3K/AKT signaling by inhibiting PTEN pathway to induce osteosarcoma cell proliferation (**Figure 6F**) (217). miR-196a transfection also decreased PTEN expression in osteosarcoma cells and led to enhanced phosphorylation of PI3K and AKT. miR-196a should therefore be an oncogene in osteosarcoma. miR-196a overexpression affected MG63 and U-2OS by regulating the PTEN/PI3K/AKT pathway cell apoptosis, cell cycle, and proliferation (222). In osteosarcoma, there are many other microRNAs with similar mechanisms of action to the above microRNAs, including miR-19a-3p (214), miR-216 (215), miR-208a-3p (216), miR-214 (58), miR-9-5p (218), miR-620 (219), miR-130a (56), miR-21 (166), miR-21 (223), miR-221 (94), miR-214 (224), miR-92a (170), miR-214-3p (226), miR-93 (227), miR-1908 (60), miR-21 (228), miR-17 (95), miR-148a (171), miR-181b (230), miR-21 (231), miR-214 (232), miR-532-5p (176), miR-21 (236), miR-744 (237), which were found to be highly expressed in osteosarcoma tissues and osteosarcoma cell lines, all of which can target PTEN, bind to the 3,URT of PTEN and reduce its protein expression. Therefore, the aberrant activation of the PI3K/AKT pathway ultimately results in the facilitation of malignant biological processes such as proliferation, migration, invasion, cell cycle regulation, autophagy and apoptosis in osteosarcoma. In addition, overexpression of miR-181a-5p (55), miR-214 (58) and miR-221 (94) also significantly increased the sensitivity of osteosarcoma cells to some chemotherapeutic drugs, such as ADR and CDDP, which provides new hope to address the current problem of drug resistance in osteosarcoma. In terms of clinical characteristics and patient prognosis, miR-130a (56), miRNA-21 (166), miR-92a (170), miR-148a (171) and miR-532-5p (176) were significantly correlated with the clinical characteristics of patients, and the analysis of clinical and pathological data of patients with osteosarcoma revealed that The high expression of miR-148a (171) correlated with the clinical stage of patients, and the high expression of miR-130a (56)and miR-532-5p (176) correlated with the clinicopathological characteristics, TNM stage, and distant metastasis of patients. The high expression of miR-21 (166) correlated with the TNM stage and late diagnosis of patients, and miR-92a (170) high expression was not only correlated with clinical staging but also with T staging and histological differentiation. In terms of prognosis, high expression of miR-130a (56), miR-21 (166), miR-148a (171) and miR-532-5p (176) was found to lead to significantly lower overall and disease-free survival of patients, and these findings are sufficient to suggest that miR and PTEN/PI3K/AKT signaling pathway interactions lead to patient prognosis. The overall point is that higher microRNA expression of these oncogenic factors that directly target PTEN in osteosarcoma has a greater impact on the PI3K/AKT pathway, which ultimately leads to higher malignancy and worse prognosis for patients with osteosarcoma.

**Figure 7:**
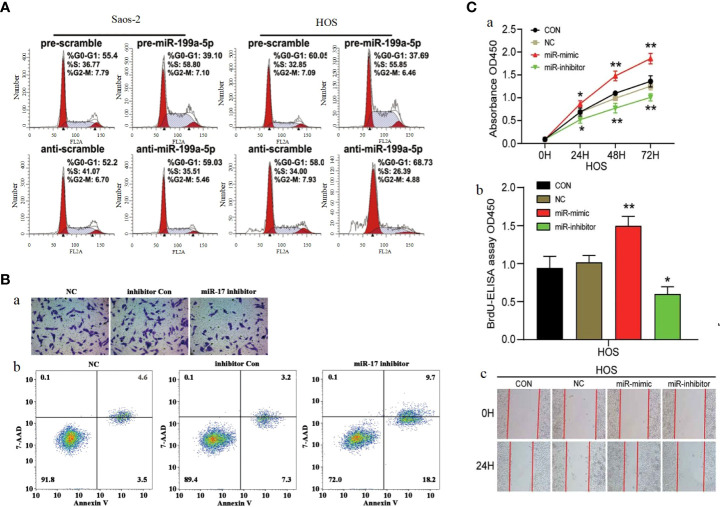
**(A)** Cell cycle analysis of Saos-2 and MNNG/HOS cells after transfection with pre/anti-miR-199a-5p or the corresponding control (221). **(B)** Knockdown of miR-17 inhibited cell proliferation, migration and invasion in OS cells.(a) Cell migration was detected using Transwell assay. **(b)** Flow cytometry demonstrated that knockdown of miR-17 induced cell apoptosis (220). **(C)** MiR-23b-3p promoted the cell viability, proliferation and migration in OS. **(A)** Cell viability was detected by CCK-8 assay. **(B)** The effect of miR-23b-3p on cell proliferation was detected by BrdU-ELISA. **(C)** Wound healing assays (184). *p < 0.05, **p < 0.01.

P27 (also known as CDKN1B) is a cyclin-dependent kinase inhibitor. As a downstream molecule of PI3K/AKT pathway, P27 is regulated by PI3K/AKT pathway, and the abnormal change of p27 significantly affects cell proliferation and cell cycle, making it a target to be considered in cancer therapy (246, 247). Several studies have confirmed that microRNAs can affect the function of PI3K/AKT pathway by influencing P27 and thus, for example, miR-199a-5p (**Figure 7A**) (221), miR-802 (225) and miR-25 (234) can act on P27 to affect its expression by the mechanism of miR-199a-5p, miR-802 and miR-25 can directly P27 the 3-UTR binding of mRNA and mediate a decrease in P27 protein levels, thus stimulating OS cell cycle progression. MiR-106b-5p was found by Chuan He et al. to cause a significant increase in the percentage of G0/G1 phase cells and a decrease in S and G2/M phase cells The number decreased, suggesting that miR-106b-5p blocks cell cycle progression by blocking osteosarcoma cells in G0/G1 phase.

CDKN1A is a direct target gene of miR-106b-5p, and the expression of miR-106b-5p exhibits a negative correlation with that of CDKN1A. CDKN1A is a key protein that can interact with CDK and is involved in the regulation of cell cycle progression, proliferation, survival, motility and senescence by binding to CDK and/or its subunits (248) (249).. In addition, the oncogenic effect of miR-95-3p can also be achieved by acting on CDKN1A. In terms of clinical features, the high expression of miR-95-3p is positively correlated with the clinical stage of patients (168). MiR-17 is highly expressed in osteosarcoma cells, and Dajiang Wu et al. have demonstrated miR-17 was involved in the development of osteosarcoma by targeting the SASH1/PI3K/AKT pathway leading to OS cells proliferation, migration and inhibition of apoptosis (**Figure 7B**) (220). As an oncogenic factor in osteosarcoma, miR-23b-3p is highly expressed in osteosarcoma tissues and OS cell lines. MiR-23b-3p directly targets VEPH1 to inhibit the activation of PI3K/AKT pathway and significantly promoted the viability, proliferation and migration of OS cells (**Figure 7C**) (184). miR-18a-5p overexpression expression inhibits SOCS5 attenuated the effects of FER1L4 overexpression on OS cell apoptosis and the expression levels of PI3K, AKT, Twist1, N-cadherin and Vimentin. Downregulation of miR-18a-5p promotes SOCS5 can PI3K/AKT pathway activation (233). MiR-214 was shown to downregulate PHLDA2 expression by targeting 3’-UTR, by Yi Li et al. High levels of miR-214 were found in osteosarcoma tissues by qPCR analysis and positively correlated with lung metastasis. Knockdown of miR-214 significantly augmented the radiosensitivity of osteosarcoma cells both *in vitro* and *in vivo*. Further investigation revealed that PHLDA2 expression was markedly suppressed, which was significantly associated with the experimental outcomes. MiR-214 modulates PHLDA2 expression to activate the PI3K/AKT pathway. Collectively, our findings suggest that the miR-214/PHLDA2/AKT axis (182). Yufeng Liu et al. found that miR-373 was significantly overexpressed in spinal cord OS tissues and OS cell lines. Overexpression of miR-373 can enhance the malignant degree of osteosarcoma cell lines, which is due to its ability to affect the expression of p53 and its downstream target genes, leading to abnormal activation of PI3K/AKT-Rac1-JNK signaling pathway. In addition, miR-373 expression was also significantly correlated with TNM stage and size of osteosarcoma. The cancer-promoting mechanism and clinical characteristics of miR-373 are expected to provide new ideas for the treatment of spinal osteosarcoma (173).

## 5 Potential biomarkers in the microRNA/PI3K/AKT axis

Despite recent advances in medical technology, early diagnosis, further effective treatment and prognosis of OS remain a serious challenge, and finding new OS biomarkers may be a feasible approach. There is increasing evidence that PI3K/AKT pathway-associated microRNAs are closely associated with OS progression. Micrornas related to PI3K/AKT pathway may be of great significance in the early diagnosis, effective treatment and prognosis of osteosarcoma, and are expected to become potential biomarkers (**Table 4**). In this section, we will discuss in detail the possible potential applications of microRNAs related to PI3K/AKT pathway in clinical practice.

**Table 4:** Potential biomarkers in the microRNA/PI3K/AKT pathway in OS.

MicroRNA	Expression	Clinical relevance	Ref
miR-3927-3p	Downregulated	Diagnostic	(162)
miR-181a-5p	Upregulated	Treatment	(55)
miR-106	Upregulated	Diagnostic	(163)
miR-183-5p	Downregulated	Diagnostic, Prognosis	(164)
miR-130a	Upregulated	Diagnostic, Prognosis	(56)
miRA-340-5p	Downregulated	Diagnostic, Prognosis, Treatment	(165)
miR-21	Upregulated	Diagnostic	(166)
miR-133b	Downregulated	Diagnostic	(167)
miR-95-3p	Upregulated	Diagnostic	(168)
miR-506-3p	Downregulated	Prognosis	(159)
miR-22	Downregulated	Treatment	(185)
miR-200c	Upregulated	Prognosis	(169)
miR-221	Upregulated	Treatment	(94)
miR-29b	Downregulated	Treatment	(187)
miR-92a	Upregulated	Diagnostic, Prognosis	(170)
miR-615	Downregulated	Diagnostic, Prognosis	(59)
miR-1908	Upregulated	Diagnostic, Prognosis	(60)
miR-100	Downregulated	Treatment	(194)
miR-148a	Upregulated	Diagnostic, Prognosis	(171)
miR-449a	Downregulated	Diagnostic, Prognosis	(172)
miR-373	Upregulated	Diagnostic	(173)
miR-141-3p	Downregulated	Diagnostic, Prognosis	(174)
miR-142	Downregulated	Treatment	(199)
miR-499a-5p	Downregulated	Diagnostic	(175)
miR-532-5p	Upregulated	Diagnostic, Prognosis	(176)
miR-128	Upregulated	Treatment	(177)
miR-29c-3p	Downregulated	Diagnostic, Prognosis	(178)
miR-520c-3p	Downregulated	Diagnostic	(179)
miR-149-5p	Downregulated	Prognosis	(180)
miR-195-5p	Downregulated	Diagnostic, Prognosis	(181)
miR-214	Upregulated	Diagnostic, Treatment	(182)

### 5.1 Diagnosis

It is well known that timely and accurate diagnosis of osteosarcoma is the key to the treatment of osteosarcoma, and also an important guarantee to improve the prognosis of patients with osteosarcoma. Identifying appropriate biomarkers has been a challenge in OS research. MicroRNAs related to the PI3K/AKT pathway have been reported to be used to aid in the diagnosis of many cancers. A comparison of tumor tissues from patients with osteosarcoma and the corresponding paracancerous tissues revealed that miR-106 (163), miR-130a (56), miR-21 (166), miR-95-3p (168), miR-92a (170), miR-1908 (60), and miR-148a (171), miR-373 (173), miR-532-5p (176), miR-128 (177) and other PI3K/AKT pathway-related microRNAs all showed high expression in osteosarcoma, and then the clinical and pathological data of these patients were analyzed and studied in depth, and their expression was correlated with clinical stage and TNM stage were positively correlated. While miR-183-5p (164), miR-133b (167), miR-615 (59), miR-499a-5p (175), miR-29c -3p (178), miR-520c-3p (179), miR-195-5p (181), miR 141-3p (174) and other PI3K/AKT pathway-related microRNAs all showed low expression in osteosarcoma, and their expression was negatively correlated with clinical stage and TNM stage. Therefore, these microRNAs can be used as valuable biomarkers for diagnosing the clinical stage and TNM stage of OS patients. In addition, miR-615 (59), miR-532-5p (176), also correlated with lymph node metastasis, and the low expression of miR-615 in osteosarcoma or high expression of miR-532-5p suggested the possibility of lymph node metastasis, so miR-615 and miR-532-5p are useful for diagnosing lymph node metastasis Therefore, miR-615 and miR-532-5p are meaningful for diagnosing lymph node metastasis. In terms of pathological diagnosis, it was found that low expression of miR-130a (56) and miR-449a (172) or high expression of miR-340-5p (165) suggested the possibility of malignant pathological features, so this kind of PI3K/AKT pathway-related miRNA has a certain supporting role for pathological diagnosis. However, we found that due to the insufficient stability of micrornas related to PI3K/AKT pathway, poor correlation between micrornas and the early stage of osteosarcoma, few clinical studies and other reasons, their use as biomarkers for the early diagnosis of osteosarcoma is limited. Therefore, more in-depth studies are needed to solve these problems.

### 5.2 Treatment

Currently, the treatment of OS mainly includes aggressive surgical resection, systemic chemotherapy and targeted radiation therapy. Although such a comprehensive treatment approach has achieved certain results, the resistance to chemotherapy and the insensitivity to targeted radiation therapy also lead to unsatisfactory treatment results and prognosis for many patients. microRNA-based targeted therapeutic strategies may provide new ideas for the development of OS therapy. Several studies have shown that microRNAs can modulate the chemosensitivity and radiosensitivity of OS by directly or indirectly interacting with PI3K/AKT pathway. Cisplatin is the traditional first-line chemotherapeutic agent for OS (250). Therefore, increasing the sensitivity of osteosarcoma cells to cisplatin is very meaningful for the treatment of osteosarcoma, and it was found that miR-181a-5p (55), miR-214 (58), miR-221 (94), miR-128 (177) and other microRNAs related to the PI3K/AKT pathway in osteosarcoma cells They are pro-oncogenic factors, and by inhibiting their expression, the sensitivity of osteosarcoma cells to CDDP can be improved. In contrast, miR-22 (185), miR-29b (187), miR-100 (194), miR-142 (199), miR-497 (200), miR-340-5p (208) and other microRNAs related to PI3K/AKT pathway showed bottom expression, and by promoting their expression, the sensitivity of osteosarcoma cells to CDDP could be improved. In addition, Ze-Yu Sun found that promoting miR-152 overexpression also improved OS sensitivity to gemcitabine (207). These findings are very important guidelines for our in-depth understanding and study of chemotherapeutic drug resistance in microRNA/PI3K/AKT pathway osteosarcoma cells, and we can target these microRNAs to find possible solutions to the resistance of osteosarcoma cells to pharmacotherapeutic drugs. In radiation therapy, Yi Li et al. demonstrated that upregulation of miR-214 significantly reduced the radioresistance of osteosarcoma cells, while upregulation of miR-214 increased its targeted binding to the 3’-UTR region of PHLDA2, resulting in decreased PHLDA2 expression and enhanced the radiosensitivity of osteosarcoma cells and a mouse xenograft model. This study suggests that it is feasible to enhance the radiosensitivity of sarcomas *via* the microRNA/PI3K/AKT axis (182). The treatment of osteosarcoma with mirnas associated with PI3K/Akt signaling pathway can be conducted from two aspects. One is to design drugs for new targets. For example, Formononetin (FN) can induce apoptosis of osteosarcoma cells by targeting miR-214-3 p and inhibit proliferation of osteosarcoma cells (226). The second is to conduct research on existing anti-osteosarcoma drugs to find miRNAs that can promote the effect of existing anti-osteosarcoma drugs, improve the sensitivity of existing drugs and toxicity against tumor cells.

### 5.3 Prognosis prediction

Early prognostic information is important in making treatment decisions for patients with osteosarcoma. In recent years, there has been increasing evidence that microRNAs associated with the PI3K/AKT pathway may have important prognostic value. miR-130a (56), miR-200c (169), miR-92a (251), miR-148a (171), miR-532-5p (176) and miR-128 (177) are promoters of osteosarcoma, and their high expression was found to be negatively correlated with the overall survival of patients. While miR-183-5p (164), miR-340-5p (165), miR-506-3p (159), miR-615 (59), miR-449a (172), miR-141-3p (174), miR-29c-3p (178), miR-149-5p (180) and miR-195-5p (181) low expression was negatively correlated with overall survival of Patients with Osteosarcoma. Among them, high expression of miR-130a (56), miR-92a (170) and miR-128 (177) also showed a negative correlation with the disease-free survival of patients. The expression profiles of these microRNAs associated with the PI3K/AKT pathway are of great interest as early prognostic information.Therefore, efforts need to be made to identify PI3K/AKT related miRNAs that are prognostic early in the development of osteosarcoma, which would be of great benefit to patients.

## 6 Conclusions and future perspectives

The PI3K/AKT pathway is heavily involved in the development of osteosarcoma and involved in various cellular functions of osteosarcoma cells, including the regulation of proliferation, migration, invasion, apoptosis, autophagy, angiogenesis, EMT, chemotherapy resistance and aerobic glycolysis during OS progression. MiRNAs are also involved in osteosarcoma caused by abnormal PI3K/AKT pathway. Identification and utilization of aberrant expression patterns of miRNAs associated with the PI3K/AKT pathway will facilitate clinical applications, including diagnosis, treatment, and prognosis of patients with osteosarcoma.

However, the current understanding of miRNA and PI3K/AKT pathway in the academic community is limited. The research on the mechanism of miRNA in osteosarcoma is mostly limited to a single or a few miRNA, and most of the related experiments are carried out in animals, human tissues and osteosarcoma cell lines, and the clinical practice research is relatively rare. This has limited our understanding of the miRNA/PI3K/AKT pathway. It also limits our knowledge and understanding of the interrelationship between miRNA/PI3K/AKT pathway and osteosarcoma development. In addition, MiRNA related to PI3K/AKT pathway are also potential therapeutic biomarkers for osteosarcoma. However, there are still many problems with miRNA as biomarkers for diagnosis, treatment and prognosis of osteosarcoma patients. For example, there are no drugs related to miRNA used for large-scale clinical treatment of osteosarcoma. As diagnostic biomarkers for osteosarcoma, most mirnas cannot be used as diagnostic markers for early osteosarcoma, such as miR-130a (56), miR-133b (167), miR-499a-5p (175) etc., which can only show diagnostic role in the late stage of osteosarcoma. Therefore, the significance of miRNA as biomarkers is limited at present.

MiRNA negatively or positively regulate the progression of osteosarcoma by directly or indirectly interacting with PI3K/AKT pathway. We can enhance the expression of repressor microRNA in osteosarcoma cells or inhibit the expression of pro-cancer microRNA in osteosarcoma cells to control cancer progression. For example, overexpression of MicroRNA-26a and miR-29a-3p can significantly reduce the proliferation, migration and invasion ability of OS (138, 210). MiR-524 knockdown can significantly inhibit the proliferation of osteosarcoma cells (245). MiRNA also affect the resistance of osteosarcoma cells to chemotherapeutic drugs. By inhibiting the expression of miR-181a-5p (55), miR-214 (58), miR-221 (94) and miR-128 (163), the sensitivity of osteosarcoma cells to cisplatin (CDDP) can be improved. Promoting the expression of mirnas such as miR-22 (208) can improve the sensitivity of osteosarcoma cells to cisplatin (CDDP) and enhance the therapeutic effect of chemotherapy drugs. Therefore, strengthening the research and investigation of the mechanism of miRNA involved in anti-tumor drug resistance will provide clinical support for strategies to overcome drug resistance, and should provide more effective new insights into the development of therapeutic methods involving miRNA. Further understanding of the structure and function of miRNA associated with PI3K/AKT signaling is also needed. In addition, further studies are needed to confirm the interaction between miRNA involved in the PI3K/AKT pathway and their related mechanisms. In the clinical application and dissemination of new therapies targeting miRNA/PI3K/AKT pathway, there are problems such as unclear indications and contraindications, unclear side effects, and imperfect coping strategies. For diagnosis and prognosis, we wanted to find a stable, easy to detect, sensitive, and specific expression of PI3K/AKT pathway related miRNA in the early stage of osteosarcoma. The ultimate goal is to translate the research results of miRNA/PI3K/AKT pathway in osteosarcoma into clinical practice, and to use these targets to develop various effective antitumor drugs.

## Author contributions

YX and YY: These authors contributed equally to this work and share first authorship. All authors contributed to the article and approved the submitted version.
